# Treatment Satisfaction and Quality of Life among Type 2 Diabetes Patients: A Cross-Sectional Study in West Bank, Palestine

**DOI:** 10.1155/2020/1834534

**Published:** 2020-08-25

**Authors:** Maher R. Khdour, Heba B. Awadallah, Dua'a H. Al-Hamed

**Affiliations:** ^1^Faculty of Pharmacy, Al-Quds University, Abu Deis, PO Box 20002, Jerusalem, State of Palestine; ^2^Pharmacy Department Ramallah & Al-Bireh Health Directorate, West Bank, State of Palestine

## Abstract

**Objectives:**

This study had the goal of evaluating the role of treatment satisfaction among diabetic patients in the context of health-related quality of life (QoL) and medication adherence.

**Methods:**

This study, which utilized a cross-sectional design, was conducted at the Primary Healthcare Unit in the Ministry of Health in Ramallah between Feb. and May 2019. Medication adherence was evaluated with the 4-item Morisky Green-Levine (MGL) questionnaire, treatment satisfaction using the Treatment Satisfaction Questionnaire for Medication version 1.4 (TSQM 1.4), and health-related quality of life with the European Quality of Life scale (EQ-5D-3L).

**Results:**

Study participants consisted of 380 diabetic patients, of which 220 (57.9%) had high adherence to their medications and 160 (42.1%) had low adherence. Based on the classification of patient responses among the EQ-5D domains, pain/discomfort was the most influenced dimension, with 173 patients (36.1%) reporting problems, (36.1%). Also prominent were anxiety and depression (128 patients, 33.7%) and Mobility (115 patients, 30.3%). A significant relation was observed between QoL and treatment satisfaction (73.8 vs. 69.8; *P* = 0.016). Treatment satisfaction also had a significant association with the anxiety domain (39.4 vs. 28.7; *P* = 0.031).

**Conclusion:**

Participants expressed moderate satisfaction with their treatments; more satisfied patients showed greater medication adherence and had better QoL. **A**nxiety has been shown to be associated with reduced medication adherence and lower QoL.

## 1. Introduction

The chronic progressive disease diabetes mellitus (DM) is associated with elevated blood glucose level (hyperglycemia) caused by impaired insulin production, impaired insulin function, or both (1). Prolonged hyperglycemia can lead to microvascular complications that impact the eyes, kidneys, and nerves; it also leads to heightened risk of macrovascular complications including CVD, peripheral artery disease, and stroke (2). Worldwide, the burden of diabetes is increasing due to the universal increase in the prevalence of obesity and unhealthy lifestyles (3). The worldwide prevalence of diabetes was about 8% in 2011 and is predicted to rise to 10% by 2030, making DM a major cause of death globally (4). In recent years, the prevalence of DM in Palestine has increased significantly (5), leading to increased risk of complications, rates of morbidity and mortality, and spending on health care. Management of diabetes and its complications by patients needs to be improved (6). However, successful control of DM by patients requires a complex and long-term approach that necessitates a great deal of commitment from patients. Diabetic patients should eat healthy food, be physically more active, and do frequent self-monitoring of their blood sugar (7). The degree to which a patient implements lifestyle changes follows a diet or takes medication in keeping with the instructions of their health care provider is referred to as adherence (8).

Treatment satisfaction, described as the cognitive evaluation of whether a treatment meets or exceeds the patient's personal subjective expectations (9), is a key factor in achieving good adherence to medication (10). It is one of several patient-reported outcomes (PROs), which are important to health providers for realizing patient viewpoints on their current medications (11); these can be applied to evaluate how disease and medication impact patient well-being, functioning, and everyday life (12). As a class, PROs measure endpoints derived directly from patient reports of their perceptions, including self-reported symptoms, functional status, and health-related quality of life (HRQoL) (13).

Surveying treatment satisfaction in particular has broad implications for enhancing health-related QoL of diabetic patients (14), for whom improving quality of life is critical. Notably, HRQoL concerns health aspects as well as general QoL; it is the patient's understanding of the impact of their disease or treatments on their QoL. These two concepts, QoL and HRQoL, are used interchangeably (15).

Few studies have been conducted to assess the association between health quality of life and treatment satisfaction among patients with type 2 DM in West Bank. The importance of this study emerged from that the determinants of treatment satisfaction in DM are poorly understood. Better knowledge of these determinants could provide clues to improving QoL in patients with DM. A greater awareness of the factors influencing HRQoL and treatment satisfaction would give physicians useful information into the multidimensional effects of this complex disease and help avoid or minimize the incidence of complications.

## 2. Study Aim

This study aimed at evaluating HRQoL among a sample of T2DM patients and its relationship with their treatment satisfaction and medication adherence. This study is one of the few studies that assess these relationships for diabetic patients in Palestine and in the Arab world in general.

## 3. Methods

### 3.1. Study Design and Site

This cross-sectional study was carried out at the Primary Healthcare Unit in the Ministry of Health in Ramallah between Feb. and May 2019.

### 3.2. Ethical Approval

Ethical approval for the study was provided by the Research Ethical Committee at Al-Quds University (REF NO. 80/REC/2019). Data collection for this study was approved by the Palestinian Ministry of Health in Ramallah (REF NO. ADM295408). Each patient was provided with an explanation of the study. Patients were informed that they could refuse to participate, discontinue their participation at any point, and refuse to answer any questions. Each patient gave a verbal consent form before the beginning of the questionnaire completion.

### 3.3. Sample Size and Participants

The sample size was estimated based on the worldwide prevalence of diabetes among adults was 7% in 2019 according to Cochran's Formula used to calculate the sample size: *n* = [(*Z*_*α*/2_)^2^ *p*(1 − *p*)/*d*^2^].

The prevalence of diabetes mellitus estimated to be 10% in Palestine, the sample size was calculated to be 338 patients with diabetes. A total of 400 diabetic patients were targeted during the study period for the purpose of reducing errors in results and increasing the reliability of the study.

The inclusion criteria were 1—diagnosed with T2DM, 2—male or female patient >18 years old, 3—taking DM medications for >3 months (in order to ensure that the patients were aware of their medications). Only 380 of the sampled patients accepted to participate and gave a verbal consent form. After giving consent, the questionnaires were completed while patients waited for their appointments. It took 15 to 20 min to interview a participant.

### 3.4. Measurement

There were four parts to the survey instrument used for this study: demographic and clinical information obtained directly from patients and their medical files; assessment of medication adherence using the Morisky Green-Levine (MGL) questionnaire; assessment of treatment satisfaction with the Treatment Satisfaction Questionnaire for Medication 1.4 (TSQM1.4); and assessment of HRQoL with the EuroQol 5 Dimension three-level scale (EQ-5D-3L). All questionnaires we used are validated and demonstrated to have reliability in with the context of chronic disease; furthermore, the Arabic versions are suitable and acceptable to be used in the Arab World.

The MGL is a four-item questionnaire for which all responses are dichotomous (No = 0 and Yes = 1). Scores are added together for a total ranging between 0 and 4, with (0) = high adherence, (1, 2) = medium adherence, and (3, 4) = low adherence (16). Its internal consistency is moderately acceptable (Alpha = 0.61).

The TSQM 1.4 is a 14-item instrument that evaluates four domains relating to treatment satisfaction: (1) effectiveness (questions 1–3), that is, condition prevention or treatments, symptom relief; (2) side effects (questions 4–8), that is, interference with physical and mental functioning, mood, or emotions; (3) convenience (questions 9–11), that is, ease of medication use and planning, frequency of medicine use; and (4) overall satisfaction (questions 12–14). For each domain, a total score of 0 to 100 was calculated according to the direction of the instrument's authors (17, 18). Higher scores represent greater satisfaction for a particular domain (10). The internal consistency is Alpha = 0.92 for effectiveness, 0.97 for side effects, 0.86 for convenience, and 0.89 for global satisfaction (19).

The European Quality of Life questionnaire is a generic, valid, and reliable instrument that consists of the five dimensions most important to patients, of which four are physical domains and one is psychological. There are two parts to the EQ-5D-3L: the EQ-5D descriptive system and the EQ visual analogue scale (EQ-VAS). The descriptive system consists of five modalities: (1) mobility; (2) self-care; (3) usual activities; (4) pain/discomfort; and (5) anxiety/depression. Patient responses to each item select the statement most reflective of their health state (10): (1) no problems; (2) slight problems; or (3) considerable problems. The internal consistency and validity of the EQ-5D-3L were determined in this study (Alpha = 0.84).

### 3.5. Data Analysis

All analyses were carried out with Statistical Package for the Social Sciences (SPSS) version 22.0. Mean ± SD was used to express continuous variables. The relationships between categorical variables were measured using Chi-squared tests, and the associations between means of continuous variables were measured using independent *t*-tests. For *P* values ≤ 0.05, results were considered to be statistically significant.

For all numerical data, descriptive statistics consisted of means and standard deviations. Categorical data were summarized as frequencies and percentages.

## 4. Results

### 4.1. Patients' Characteristics

A total of 400 patients were met during the study period, 20 patients were excluded, and they did not meet the inclusion criteria for our study. The patients mean age was 52.97 ± 13.95, with 57.9% males. The duration of the first diabetes diagnosis for approximately 30% of patients was at least 10 years. Most of the patients (78.2%) were obese. Most patients (76.6%) were married and (35.5%) had school tertiary level. Regarding the patient's lifestyle, (65.8%) of patients never smoke. Hypertension (51.6%) of patients was the most common comorbid condition affecting patients. Hyperlipidemia was a major complication of T2DM in (14.5%) of patients, while CVD affected (2.4%) of patients. Retinopathy was the most common minor complication affecting (27.6%) of patients in Table 1.

The majority of the patients (82.1%) reported taking Metformin as a monotherapy. (29.5%) reported taking Metformin plus Glimepiride as a combination therapy, while (0.8%) reported taking Metformin plus Vildagliptin plus Glimepiride, Metformin plus Dapagliflozin plus Sitagliptin, Metformin plus Dapagliflozin plus Vildagliptin and Metformin Dapagliflozin plus Glimepiride plus Sitagliptin as a combination therapy shown in Table 2.

### 4.2. Adherence Level

**Figure 1:**
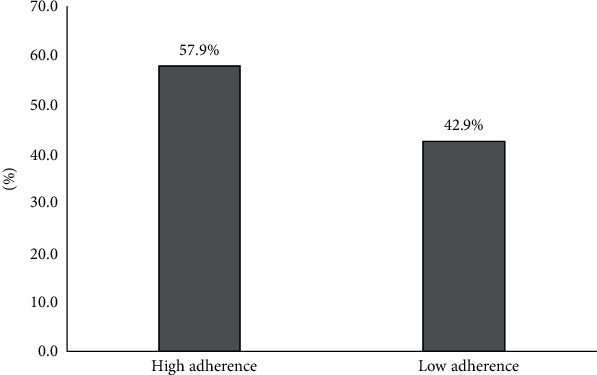
Classification of the study participants according to their adherence level.

According to the MGL questionnaire, 220 (57.9%) patients had a high adherence level and 160 (42.1%) had a low adherence level (Figure 1).

### 4.3. Quality of Life (QoL)

**Figure 2:**
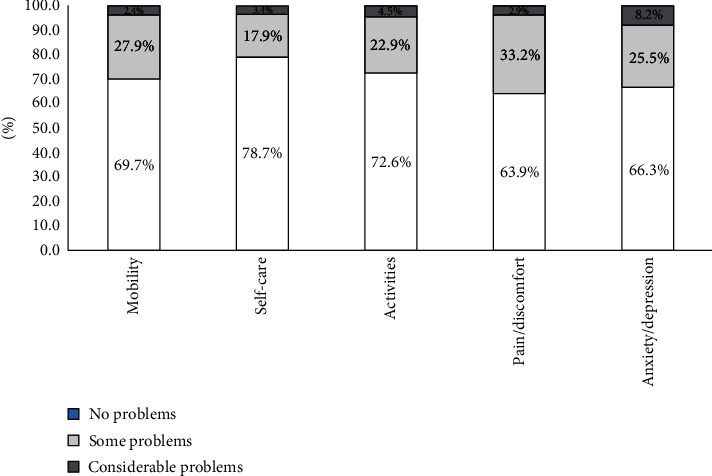
Classification of patient's response to the EQ-5D modalities. Notes: light segments, no problems; gray segments, some problems; black segments, considerable problems.

The classification of the three different response modalities for EQ-5D five dimensions is presented in Figure 2. Pain/discomfort were the most influenced dimensions (173 patients reported problems, 36.1%), anxiety and depression (128 patients reported problems, 33.7%), and the mobility (115 patients reported problems, 30.3%).

### 4.4. EQ-VAS Scores in relation to Level of Adherence

The percent of Adherent patients with a VAS score (75-83) is 66%, (62.1%) is the percent of adherent patients with a VAS score (84-100).

**Figure 3:**
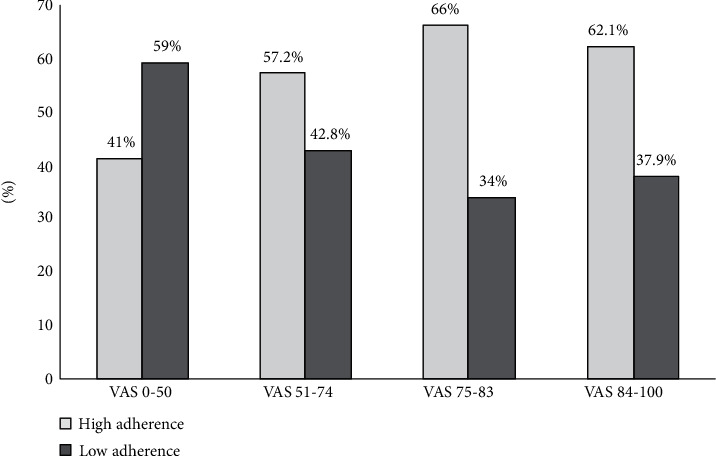
Distribution of patient's EQ-VAS scores according to their Adherence level. Notes: light segments, high adherence; dark segments, low adherence. EQ-VAS scores are divided into quartiles: 1st quartile: 0–50; 2nd quartile: 51–74; 3rd quartile: 75–83; 4th quartile: 84–100.

Patients with high adherence to medication had significantly higher VAS scores that indicated good quality of life compared to patients with low adherence to their medication as shown in Figure 3.

### 4.5. Treatment Satisfaction in relation to Quality of Life

**Figure 4:**
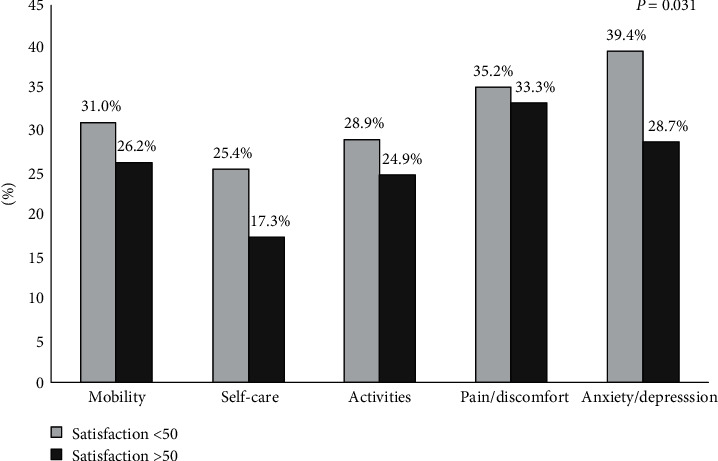
Classification of TSQM response domains for the EQ-5D.Notes: data are presented as the percentages of patients confirming some or considerable problems on each dimension of the EQ-5D. Grey columns, TSQM general satisfaction score <50; black columns, TSQM general satisfaction score >50.

Results from (Figure 4) indicated that patients with higher treatment satisfaction > 50 had lower problems in EQ-5D domains (Mobility, activities, self-care, pain and discomfort, and anxiety and depression); this results indicated more satisfied patients had a better Quality of life.

Significance association between anxiety and depression and treatment satisfaction (*P* = 0.031). More satisfied patients with their treatment reported significantly better change in anxiety and depression domain compared with not satisfied patients.

In the more satisfied patients, the overall EQ-VAS score was significantly higher (73.8 ± 15.09 vs. 69.8 ± 15.88; *P* = 0.016; Student's *t*-test); this indicated a better QOL (Table 3).

## 5. Discussion

The purpose of this cross-sectional survey is to measure the patients' HRQoL and its relation with treatment satisfaction and the relationship between QoL and adherence among a selected group of patients with diabetes mellitus in Palestine. The result in our survey showed that most of the participants (57.9%) had a high adherence level and (42.1%) had a low adherence level. This result is similar to results from other studies on adherence among diabetic patients using the same method of adherence assessment, where the adherence rate was reported to be 49.3% (20). In general, among patients with diabetes, the medication adherence level ranges from 36 to 93 (21). In contrast to our study, other studies showed lower rates of adherence (22).

More satisfied patients with their treatments reported a strong HRQoL in our study. In addition, the study population had a positive relationship between treatment satisfaction and HRQoL. Other studies conducted in Palestine about diabetes showed that there is a low connection between treatment satisfaction and HRQol. Other Dutch study revealed a low relation between treatment satisfaction and HRQOL and indicated that treatment satisfaction and HRQOL are two fairly different incidences (23).

In our study, most of the participants reported problems with pain/discomfort (36.1%) and anxiety/depression (33.7%) than other dimensions of mobility (30.3%). Our finding is comparable to previous studies. In a study from China involving type 2 diabetics, pain/discomfort was also the most frequent in several other studies among the five EQ-5D domains. While diabetes does not cause pain directly, its treatments and complications, such as injections of insulin, infections, and wounds and cuts that are slow to heal, healing, can cause pain. Anxiety and depression is the second domain EQ-5D after pain and discomfort that the patients commonly report problems (24). This finding is similar with other findings that showed poor psychological health and a high tendency to suffer from depression in patients with diabetes was related to patient's fears about complications and disease progression and frustration about inadequate therapy response (25).

In our study, (66%) of adherent patients had a VAS score (75-83), this means that patients with high adherence to medication had significantly higher VAS scores that indicated good quality of life compared to patients with low adherence to their medication.

In this survey, the result showed that there was a significant relation between HRQoL and treatment adherence, similar to previous results which suggested that the patients that had a low level of adherence was correlated with low quality of life (26). Surprisingly, a high portion of patients with poor quality of life “VAS score (0-50)” was found to adhere to their treatment; this could be explained by patients with poor quality of life had a high percentage of diabetic complications; this leads the patients to adhere more to their medications to reduce complications; another reason might be because healthcare providers are more attentive to patients with poor quality of life, which can lead to higher medication adherence.

Adherence to treatment increases the HRQOL of a patient by reducing symptoms, progression of illness, and frequency and severity of exacerbations (27).

A significant relation between QOL and treatment satisfaction was noticed in this research (*P* = 0.016), which indicated that higher satisfied patients had a higher VAS score and higher QOL (73.8 ± 15.09 vs. 69.8 ± 15.88).

Significant association between anxiety and depression and treatment satisfaction (*P* = 0.031) which means more satisfied patients had lower anxiety and depression.

Anxiety and depression were the most commonly reported problems by diabetic patients (28).

Pharmaceutical care provided by the clinical pharmacist helped in improving patient health aspect QOL; this indicated the beneficial role of pharmacist-provided counseling and education about the importance of patient's treatment satisfaction (29, 30).

### 5.1. Strengths and Limitation

The large sample size is a strong point of our research. The large included that a sample of diabetic patients allowed the survey with good statistical significance of fairly different associated factors.

### 5.2. Our Study Has Few Limitations

First, the use of a questionnaire may not always be accurate, which could lead to a bias in knowledge. Since the analysis was a cross-sectional design, it is not possible to conclude whether the various independent variables affect patient's satisfaction or vice versa. Hypoglycemia is another determinant that would have an impact on the QOL of patients and medication adherence, but this research did not take this into consideration.

### 5.3. Conclusion

In our study, most satisfied patients were found to be adherent to medication and had a good QOL. There was a significant association between adherence and QoL and a significant association between QOL and treatment satisfaction.

Special attention should be paid to patients that report anxiety or fear regarding the disease or treatment since anxiety was shown to be associated with poor adherence, lower treatment satisfaction, and QoL.

## Figures and Tables

**Table 1:** Sociodemographic and clinical patient's information.

	*N* (number of patients)	% of patients
Gender		
Male	220	57.9%
Female	160	42.1%
Age (years; mean ± SD)	52.97 ± 13.95	
BMI		
Normal	8	2.1
Overweight	75	19.7
Obese	297	78.2
Smoker		
Yes	106	27.9
No	250	65.8
Ex-smoker	24	6.3
Insurance		
Yes	358	94.2
No	22	5.8
Marital status		
Single	50	13.2
Married	291	76.6
Divorced	9	2.4
Widowed	30	7.9
Education		
Primary	12	3.2
Secondary	97	25.5
Tertiary	135	35.5
University	111	29.2
Postgraduate	25	6.6
Job		
Yes	172	54.7
No	208	45.3
Duration of disease		
3 months-1 year	58	15.3
1 year-5 years	115	30.3
6-10 years	91	23.9
>10 years	116	30.5
Family history of diabetes		
Yes	257	67.6
No	173	32.4
HA1c		
HA1C < 7 controlled	174	45.8
HA1c > 7 uncontrolled	206	54.2
Insulin		
Yes	166	43.7
No	214	56.3
Complications		
Yes	238	62.6
No	142	37.4
Retinopathy	94	27.6
Neuropathy	39	10.3
Nephropathy	20	5.3
Comorbidities		
Hypertension	196	51.6
MI	18	4.7
Stroke	17	4.5
Hyperlipidemia	55	14.5
CVD	9	2.4
Asthma	3	0.8

BMI: Body mass Index, MI: Myocardial Infarction, CVD: Cardiovascular Disease.

**Table 2:** Medications history and manner of prescribing of antidiabetic drugs.

Monotherapy	*N* (number of patients)	% of patients
Metformin	312	82.1
Glibenclamide	6	1.6
Dapagliflozin	12	3.2
Glimepiride	118	31.1
Sitagliptin	61	16.1
Vildagliptin	23	6.1
Saxagliptin	9	2.4
	*N* (number of patients)	% of patient
Metformin+Glibenclamide	6	1.6
Metformin+Dapagliflozin	3	.8
Metformin+Glimepiride	112	29.5
Metformin+Sitagliptin	52	13.7
Metformin+Vildagliptin	17	4.5
Metformin+Saxagliptin	6	1.6
Metformin+Dapagliflozin+Sitagliptin	3	0.8
Metformin+Dapagliflozin+Vildagliptin	3	0.8
Metformin+Glimepiride+Vildagliptin	3	0.8
Metformin+Dapagliflozin+Glimepiride+Sitagliptin	3	0.8

**Table 3:** EQ-VAS score correlation with treatment satisfaction.

Satisfaction	*N*	Mean	SD	SE
Overall satisfaction < less than 50	144	69.80	15.09	1.26
Overall satisfaction < more than 50	235	73.80	15.89	1.03

SD: Standard Deviation, SE: Standard Error.

## Data Availability

The data used to support the findings of this study are available from the corresponding authors upon request.
